# The Effect of Thoracolumbar Injury Classification in the Clinical Outcome of Operative and Non-Operative Treatments

**DOI:** 10.7759/cureus.12428

**Published:** 2021-01-02

**Authors:** Caitlyn J Smith, Mohanad M Abdulazeez, Mohamed ElGawady, Fassil B Mesfin

**Affiliations:** 1 Orthopaedic Surgery, University of Missouri School of Medicine, Columbia, USA; 2 Civil, Architectural, and Environmental Engineering, Missouri University of Science and Technology, Rolla, USA; 3 Civil Engineering, Missouri University of Science and Technology, Rolla, USA

**Keywords:** thoracolumbar injury classification system, posterior ligamentous complex, gray zone, computational modeling, post-treatment

## Abstract

This review assesses the validity of a biomechanical approach using finite element analysis in the Thoracolumbar Injury Classification and Severity Score System (TLICS) by addressing the “gray zone” decision discrepancy of thoracolumbar spinal injuries. A systematic review was performed using the keywords “Thoracolumbar Injury Classification” AND “finite element analysis of the spinal column” to evaluate the validity of the TLICS and finite element analysis of the thoracolumbar spinal column. Results were classified according to the main conclusions and level of evidence. Thirteen articles are included. Four of the articles evaluated the TLICS in comparison to other classification systems of thoracolumbar spinal injuries. A notable finding is that the TLICS had inconsistencies with other classification systems in the treatment of burst fractures without neurological deficits. One article evaluated the TLICS with the inclusion of magnetic resonance imaging (MRI) in the evaluation, which decreased the agreement between the suggested and actual treatment. Among the three finite element analysis studies, limited data have been published on the posterior ligamentous complex (PLC) status when an injury is suspected or indeterminate. The TLICS has been a reliable classification system in the management of single-column fractures and three-column injuries treated with surgical stabilization. Special attention to enhancing the TLICS classification system by eliminating the “gray zone” of a TLICS score of 4 is essential. Biomedical computational modeling evaluating the PLC status of indeterminate or injury suspected is needed to enhance the current TLICS system and to clarify the decision discrepancy in the “gray zone.”

## Introduction and background

Thoracolumbar (TL) fractures are the most common traumatic injuries to the spinal column. The annual incidence of TL injures in the United States is approximately 15,000; the majority of those incidents are due to high-energy trauma resulting mainly from a motor vehicle accident in younger patients [1-2]. Also, nearly 700,000 osteoporotic fractures occur annually in elderly patients [1]. Most commonly, TL injuries occur at the T10 to L2 level [3-4]. The TL junction is more susceptible to injury because there is a transition between the stiff kyphotic thoracic spine and the mobile lordotic lumbar spine [3-4]. Approximately 25% percent of TL fractures result in neurological deficit [5-6].

The four major spinal injuries are compression fractures, burst fractures, flexion-distraction injuries, and translational injuries. Numerous classification systems for thoracolumbar spine injuries have been established. However, there is no current universal acceptance of a classification system for TL spine injuries that facilitates proper communication between treating physicians and helps standardize approaches to treatment. In 1983, the three-column theory was introduced by Francis Denis to classify acute TL spinal injuries [7]. According to this theory, stability is based upon the integrity of two of the three spinal columns [7]. The Denis system may oversimplify complex fractures and may not accurately access the need for operative intervention [8].

In 1994, the AO (Arbeitsgemeinschaft für Osteosynthesefragen System) classification was introduced using a mechanistic approach to separate fractures into 53 different patterns based upon three injury categories and three tiers of subcategorization [5]. The use of 53 different fracture patterns makes routine clinical use of the classification subject to poor inter- and intra-observer agreement [9-10].

In 2005, the Spine Trauma Study Group introduced a classification system for TL injuries named the Thoracolumbar Injury Classification and Severity Score (TLICS). This score assigns numerical values to each injury based upon the morphology, neurologic status, and integrity of the posterior ligamentous complex (PLC) [8]. A patient with a TLICS score higher than five is considered a surgical candidate, and a patient with a score less than three are often non-surgical candidates. The treatment strategy of patients with a total TLICS score of four is unclear. A score of four points remains a gray zone that permits surgeons to use individual clinical judgment to determine surgical options. Another TLICS gray zone shortfall was related to the surgeon’s inability to agree on the integrity of the PLC. Definite criteria of PLC injury may be necessary because the differentiation of PLC injury between TLICS score 0, 2, and 3 is very difficult [11].

A classification system is required to facilitate effective communication between spine surgeons, to guide treatment, and to help predict the prognosis. An ideal system should be simple, comprehensive, reliable, and reproducible, with predictive outcomes. Unfortunately, most of the existing classifications have failed to fulfill the above criteria; some are oversimplified while others are too inclusive and complex for routine use. While there is no acceptance of a universal TL spinal injury classification system, it is imperative to understand the evolution of spinal injury classification.

Research significance

Despite multiple methodologies to evaluate patients with TL injuries scored four lines within the gray zone, the standardized classification and treatment of TL spine fractures remains controversial. Thus, comprehensive literature was performed in this study as an attempt to offer the most updated approaches that have been implemented with an efficiency assessment that helps in surgical decisions.

## Review

Methods

**Figure 1:**
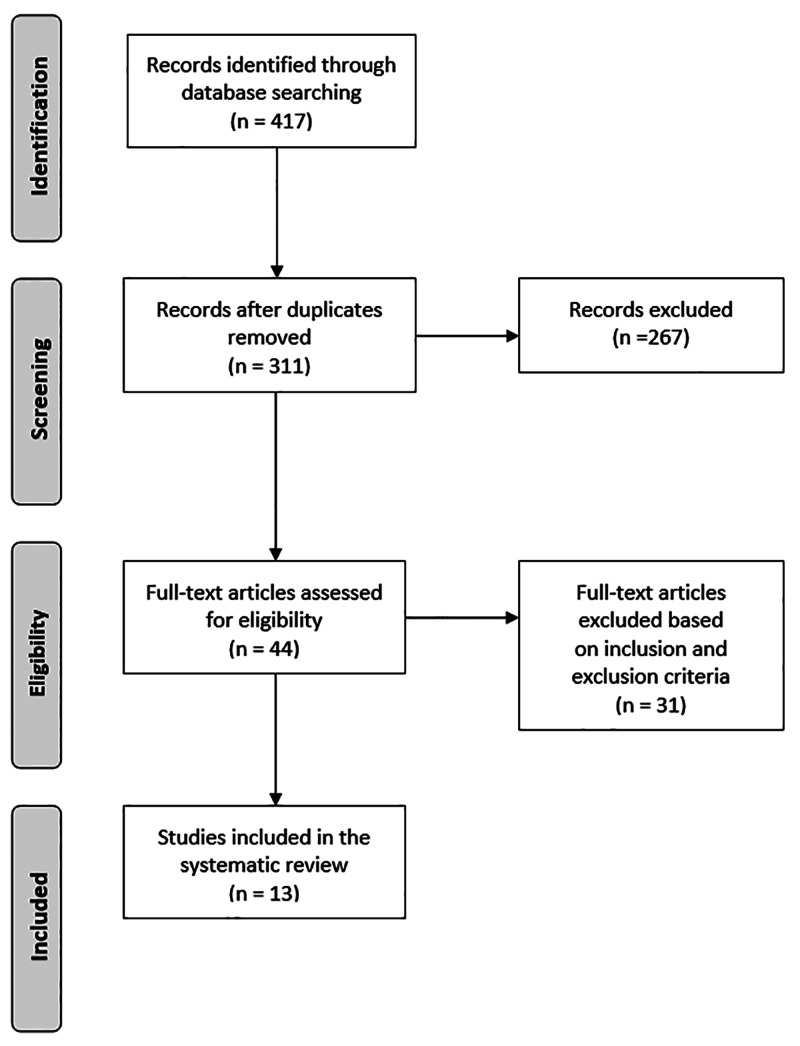
PRISMA flowchart showing record review and study inclusion Preferred Reporting Items for Systematic Reviews and Meta-Analyses (PRISMA)

A systematic literature review of available literature was performed to identify all studies dealing with accessing the validity of the Thoracolumbar Injury Classification and the finite element biomechanical models of the TL spinal column. The Preferred Reporting Items for Systematic Reviews and Meta-Analyses (PRISMA) guidelines were followed to identify the articles [12]. Using the Medline and PubMed databases, a descriptive and up-to-date review of the spine trauma literature was gathered in Table 1. The terms “Thoracolumbar Injury Classification” and “finite element analysis of the spinal column” were used in the main entry search on June 19, 2020. Two authors (C.J.S. and M.M.A.) reviewed the search results. We included only retrospective or prospective clinical publications, evaluating the reliability of the commonly used TL injury classification systems (i.e., TLICS and AO) along with biomechanical analysis studies using finite element (FE) of the thoracolumbar spine. The exclusion criteria consisted of literature reviews, case reports, personal communications, or paper presentations. The final articles were selected according to the evidence-based medicine criteria proposed by Wright et al. [13]. An electronic search yielded 417 studies. After 106 duplications were removed, 311 studies remained; 267 were excluded after review of the abstract and full-text articles, leaving 44 eligible studies. An additional 31 articles were then excluded based on additional inclusion and exclusion criteria. A total of 13 articles published between 2015 and 2020 met the inclusion criteria (Figure 1).

**Table 1:** Summary of the articles: TLICS validity and biomechanical computation modeling of thoracolumbar injuries Abbreviations: Thoracolumbar Injury Classification and Severity Scale (TLICS), Arbeitsgemeinschaft für Osteosynthesefragen System (AO System), Thoracolumbar AOSpine Injury Score (TL AOSIS), Subaxial Injury Classification and Severity Scale (SLICS), Load-sharing classification (LSC), Instantaneous Axis of Rotation (IAR), Range of Motion (ROM), Negative Predictive Value (NPV), Positive Predictive Value (PPV), FE: Finite Element, FEA: Finite Element Analysis

Study	Methodology	Objectives	Results	Conclusions
Yuksel et al., 2018 [14]	Retrospective analysis of 55 patients with TL burst fractures treated with instrumentation 55 patients with thoracolumbar burst fractures undergoing instrumentation between 2010 and 2015	Evaluate the reliability of recommendations in the surgical management of unstable TL burst fractures using the TLICS and AO TLICS and the AO System	Neurological deficits detected in 18 patients, all received a TLICS > 4; 14 patients with incomplete spinal cord injury all received a TLICS score > 4; 8/14 patients received 4 points using the AO system; 37 patients without neurological deficit received < 4 points of TLICS whereas 18/37 patients received 3 AO points, to whom AO recommends conservative treatment although they had unstable burst fractures	Compared to AO recommendation, TLICS may be more reliable in guiding surgical management of unstable TL burst fractures without neurological deficits
Dawkins et al., 2018 [15]	Retrospective review using medical records of pediatric patients with acute, traumatic thoracolumbar fractures at a single Level 1 trauma center	Evaluate the reliability of the TLICS system in pediatric patients	Mean TLICS was 3.7 ± 2.8; surgical treatment for 33.3% of patients; interrater reliability of the TLICS system had a κ value of 0.69 for the TLICS treatment; when MRI was included in the evaluation of TLICS the interrater reliability decreased, κ value decreased from to 0.57 for patients with CT only	Agreement between suggested treatment and actual treatment decreased when MRI was included in the injury assessment. Physicians should be careful when using MRI to help guide the surgical decision making
Pneumatico et al., 2016 [16]	A retrospective review of 58 patients with TL fractures (group A and B) treated conservatively were evaluated over a follow-up period of 28 months	Evaluate the effectiveness of TLICS scoring for TL spine fractures without neurological deficits and efficacy of conservative treatment in patients with TLICS 4	8.2 mean pain and 86-point functional score (group A, TLICS 1-3); 6.4 mean pain and 76 points functional score (group B, TLICS 4)	Conservative treatment of cases with a TLICS score of 4 can be safely applied and is equally as valid to those scoring <3
Guo et al., 2019 [17]	A nonlinear finite element model of T12-L1 was created to analyze the response of vertical impact load using three different mass balls to represent the different loads	Investigate the mechanism of spinal burst fracture under different energy vertical impact loads to produce a failure risk region to understand the mechanism of burst fracture and help guide clinical treatment	At low energy condition (13 J), the rigid ball rebounded rapidly; intermediate energy (30J) fracture was initiated in the vertebral foramen and left rear regions on the superior cortical bone near superior endplate of L1; high energy (56J) burst fracture occurred	The strength of the vertebral body under intermediate energy conditions was sufficient to support the impact. Burst fracture occurred at L1 only at intermediate and high energy.
Sterba et al., 2019 [18]	A finite element model of L1-L3 spinal segment and 27 sets of ligament properties was submitted to a posterior-anterior impact at three separate velocities (2.7, 5, 10 m/s)	Evaluate the effect of impact velocity and ligament properties in the lumbar region in response to traumatic flexion-shear conditions	At velocities of 2.7 and 5 m/s, a greater extent of bony injury such as volume of ruptured bone (1140, 1094, 718 mm3), lower L2 anterior displacement (2.09, 5.36, 7.72 mm), and lower facet fracture occurrence compared to impact at 10 m/s. Ligament properties influenced lower facet fracture occurrence, but no effect on bony injury initiation	These findings improve the understanding of the mechanism and load thresholds of lumbar injury
Wu et al., 2018 [19]	A FE model of the fractured TL spine was developed, and the ROM and IAR of the T12-L1 segment were measured at the fracture and sequential reduction PLC. ROM and IAR were measured under flexion, extension, lateral bending, and rotation.	Analyze the effect of consecutive ligament failure on the ROM and location of IAR of the TL spine at T12-L1 using the FE model	As ligaments of the PLC were removed sequentially, increase in ROM and IAR was reported; under flexion, failure of the SSL had the most significant influence on the change in the ROM and IAR; in extension, removal of the FCL caused the greatest shift	During injury of the thoracolumbar spine, the SSL plays a key role in allowing the PLC to maintain stability
Takano et al., 2017 [20]	A 3D image-based finite element (FE) was used to model (Th12L2) by using (CT) digital imaging and communications in medicine (DICOM) for each patient	Biomechanically analyze vertebral stress concentration in one healthy subject and one subject with osteoporotic first lumbar (L1) vertebral compression fracture by using finite element analysis (FEA)	The comparison showed that vertebral stress concentration increased with all stresses in the vertebral compression fracture models. Compression and axial rotation caused remarkable increases in stress concentration in the vertebral compression fracture models.	The FEA effectively showed that the osteoporotic subject seemed to exhibit extremely higher stresses and strains than the healthy subject under the five basic vertebral physiological motions.
Hsieh et al., 2020 [21]	A biomedical computational modeling study using five nonlinear FE models was used to assess changes in the range of motion (ROM) and stress in the spine after treating a lumbar burst fracture with the hybrid fixation method.	To evaluate the effect of cement augmentation on adjacent vertebrae after osteoporotic vertebroplasty for the treatment of TLBF. Also, biomechanical data from pedicle screws with and without bone cement augmentation in a short-segment fixation model	The hybrid fixation method of cement-augmented screw fixation (AwC-TSF-S) results in a stiffer construct and lower ROM at instrumented segments, which may also reduce the risk of fracture of adjacent vertebrae.	This study showed that the use of cement-augmented screws does not put the adjacent vertebrae at an increased risk of fracture.
Hamilton et al., 2019 [22]	Review of University of Wisconsin Hospital trauma database reviewed for tree stand injuries from 1993 to 2013	Review spine injuries due to falls from a tree stand in the context of TLICS and SLICS to access inter-user reliability and validity among neuroradiology and neurosurgery raters	Management recommendation reviewer agreement was 12/15 (80%) of SLICS and 38/52 (73%) of TLICS; operative PPV reached 100%, wide confidence interval. SLICS NPV was poor, 54%-60%	Good-to-excellent inter-rater reliability was reported in the TLICS and SLICS systems; SLICS validity was poor, whereas TLICS was reasonable for non-operative cases and moderate for operative cases
Dodwad et al., 2015 [23]	A retrospective review of 201 patients with thoracolumbar junction injuries at major academic medical centers servicing level I trauma between January 1, 2006, to March 31, 2011	Evaluate the validity of the TLICS score system by comparing the TLICS to prior management of TL injuries at their institution	98% of non-operative cases and 78% of operative cases scored by TLICS agreed with previous TL injury management at the institution	In cases of TL injuries that should be treated nonoperatively according to the TLICS score, surgeons may be more likely to surgically treat the injuries
An et al., 2020 [24]	A retrospective review of 110 patients with TL injuries hospitalized from January to September 2019	Evaluate whether TLICS and AOSIS have superiority to each other in terms of reliability of their recommendations for guiding surgical treatment	TL AOSIS matched treatment decision-making in 98.18%, and TLICS matched 87.27%; patients without neurological deficit, 12- >5, 12- 4 or 5 points, 38- <4 points according to the AOSIS system; 12 > 5, 50 <3 points, and none received 4 points according to the TLICS system	Recommendation of TL AOSIS may be more reliable than TLICS for guiding surgical treatment of burst fractures
Pishnamaz et al., 2018 [25]	Web-based intraobserver and interobserver study of 91 patients classified twice by seven board-certified spine surgeons using plain radiographs, and CT scans	Evaluate and compare the intraobserver and interobserver agreement of the LSC, TLICS, and AOSpine classification systems	Intraobserver and interobserver reliability considering LSC total score was fair, κ = 0.26/0.22; For TLICS, moderate intraobserver agreement, κ = 0.26 and fair reliability for interobserver results, κ = 0.23; AOSpine showed substantial intraobserver and interobserver agreement for fracture type, κ = 0.71/0.61 and moderate agreement for fracture subtype, κ = 0.57/0.48	Reliability of AOSpine fracture classification is superior to the TLICS and LSC
Kaul et al., 2017 [26]	Clinical and radiological data of 50 patients with a diagnosis of acute traumatic TL spine injury distributed to 11 spine surgeons at six institutions in the form of a PowerPoint presentation. Reclassification occurred after six weeks	Evaluate if the recently introduced AO Spine Classification system has better interrater and intra-rater reliability than TLICS	Moderate interrater and intra-rater reliability was seen for grading fracture type and integrity of PLC, k = 0.43 and 0.59, respectively, PLC: k = 0.47 and 0.55; Fair to moderate reliability, k = 0.29 interobserver and 0.44 intraobserver for TLICS total score; moderate interrater, k = 0.59; substantial intra-rater reliability, k = 0.68 ± 0.13 for grading fracture type regardless of subtype according to AO Spine classification	Recently proposed AO Spine classification has better reliability for identifying fracture morphology than the existing TLICS; additional studies needed to evaluate classification systems among multiple treating physicians

Results

The results reported were subdivided into findings related to the Thoracolumbar Injury Classification and finite element analysis of the thoracolumbar spinal column.

Thoracolumbar Injury Classification

Compared to the AO recommendations, TLICS may be more reliable in guiding the surgical management of unstable TL burst fractures without neurological deficits, as the AO system had recommended conservative treatment. However, the patient had an unstable burst fracture [14].

Yuksel et al. report that for patients with neurological deficits, both the TLICS and AO scoring systems recommended surgical treatment [14]. Of the 37 patients without neurological deficit, all patients received TLICS > 4, whereas 19 out of the 37 patients received an AO score between 1 to 3 points in which AO recommends conservative treatment (Table 2). However, the patients had unstable burst fractures.

**Table 2:** Comparison of thoracolumbar injury scores of patients with an unstable burst fracture Source: Yuksel et al., 2018 Abbreviations: Thoracolumbar Injury Classification and Severity Scale (TLICS), Arbeitsgemeinschaft für Osteosynthesefragen System (AO System)

TLICS (N = 55)			
	1-3	4	> 5
Neurological Deficit (N = 18)	-	-	18
Neurological Function Intact (N = 37)	-	-	37
Incomplete Spinal Cord Injury (N = 14)	-	-	14
AO System			
Neurological Deficit (N = 18)	-	-	18
Neurological Function Intact (N = 37)	18	-	19
Incomplete Spinal Cord Injury (N = 14)	-	8	6

Dawkins et al. reported that the inclusion of magnetic resonance imaging (MRI) in the evaluation changed the final TLICS score leading to a decrease in the agreement between the suggested and actual treatment [15]. In the Dawkins et al. study, when MRI was used to calculate the TLICS score, patients treated conservatively were likely to have a score suggesting surgical treatment.

In the Pneumaticos et al. (2016) study, the mean follow-up was 28 months in which conventional treatment for a TLICS score of 4; TL fractures were reported as equally valid to those scoring <3 [16].

Finite Element Analysis of the Thoracolumbar Spinal Column

Guo et al. analyzed the TL burst fractures under vertical impact loads using the FE method [17]. At high energy conditions (56 J), the burst fracture occurred on the L1 segment, and the fracture pattern was observed in clinical practice.

Sterba et al. analyzed ligament mechanical properties on the lumbar spine in posterior-anterior impact loading conditions [18]. At high velocity (10 m/s), a major or complete rupture was determined, scoring an additional three points according to TLICS leading to a total TLICS score > 5. Additionally, Wu et al. reported the significance of the supraspinous ligament (SSL), as its failure led to the greatest change in range of motion (ROM) and instantaneous axes of rotation (IAR) under flexion [19]. Thus, the SSL plays a crucial role in maintaining the stability of the thoracolumbar spine during injury.

A finite element study was performed by Wu et al., where the biomechanical role of the TL ligaments of the PLC was analyzed [19]. The study evaluated the effect of consecutive ligament failure and the role of ligaments in maintaining the stability of the injured TL spine. A consistent increase in the ROM and location of IAR as the ligaments were consecutively removed was determined by the model. A notable finding was the SSL had the greatest influence on the change in the ROM and IAR under flexion, allowing the PLC to maintain the stability of the TL spine during injury.

Finite element modeling can provide a practical understanding of compression injuries of different patient types. Takano et al. used modeling to analyze the vertebral stress concentration of a healthy subject compared to an individual with an osteoporotic L1 vertebral compression fracture [20]. Under five basic vertebral physiologic motions, higher stress, and the strain exhibited by the osteoporotic subject, finite element analysis provides a useful method to evaluate injury patterns of the spine and a comprehensive understanding of each patient’s condition, which is crucial in determining the best surgical treatment.

Hsieh et al. reported the use of a hybrid fixation method combining vertebroplasty and cement-augmented screws for securing TL burst fractures that resulted in a stiffer construct and lower stress on the pedicle screws [21]. The hybrid fixation method presented in this study showed that the use of cement-augmented screws does not increase the risk of adjacent level vertebral fracture.

Discussion

As shown in Table 1, the evidence for evaluating the validity of the TLICS is favorable in the last decade. The TLICS has been a reliable classification system in the management of single-column fractures treated conservatively, and three-column injuries (flexion/extension distraction injuries and fracture-dislocations) treated with surgical stabilization [22-23]. However, limited data have been published addressing the TLIC score of 4 or gray zone in which there is a lack of standardization of surgical or non-surgical management among treating physicians.

A notable finding is that the retrospective evaluation of the TLICS had inconsistencies with other classification systems in the treatment of burst fractures without neurological deficits. Additionally, a significant finding reported by Yuksel et al. is the treatment decision discrepancy between the TLICS and AO classification systems in unstable burst fractures (Table 2) [14]. Standardization of TL injury scores is crucial to guiding proper surgical management among treating physicians.

Despite the increased reliability of the TLICS in the management of unstable burst fractures, the Thoracolumbar AOSpine Injury Score was recommended as more reliable than the TLICS system in the treatment of burst fractures, fracture classification, and morphology [24-26]. A potential explanation for the inconsistencies is that the TLICS system is that it does not consider particular factors such as segmental kyphosis, loss of vertebral height, and degree of canal compromise for guiding surgical treatment. A limited evidence-based relationship with the patient’s outcome reported the inclusion of these factors [9]. The TLICS system does not account for these factors for guiding surgical treatment. The TLICS classification system has left an ambiguous zone for burst fractures (2 points) when evaluating the PLC as an injury suspected, or indeterminate of the PLC has scored 2 points, giving a total TLICS score of 4 points. Another probable reason for inconsistencies in the retrospective evaluation of the TLICS is according to the type of radiologic assessment, and the TLICS score can change.

While initial MRI of the spine is not standard of routine care at many trauma centers, studies have proposed that when the presence or disruption of the PLC was not clear on computed tomography (CT), an MRI would be useful. A potential explanation for the finding reported by Dawkins et al. is differences in the final PLC score as some raters may have increased the score, and some kept it the same [15]. Also, an MRI was ordered in cases in which the surgical decision-making process was not clear from CT alone.

An additional problem noted in this study is that the evaluation of TLICS validity is the lack of a gold standard for measuring the treatment of thoracic and lumbar spinal trauma (TLST). In the current literature, many studies have evaluated the outcome of specific injury patterns. Few studies have assessed the long-term patient-based outcomes in the conservative or surgical management of their injuries. However, the lack of a universally accepted standard classification system for TL injuries has limited our ability to further access the utility of the TLICS in terms of evaluating patient’s TLICS score at the time of injury and treatment and their long-term reported outcomes. Special attention to enhancing the TLICS classification system by eliminating the gray zone of a TLICS score of 4 is necessary. Biomedical computational modeling may be used on the TL spine to enhance the current TLICS classification by standardizing treatment among treating physicians.

As many researchers have proposed classification systems and extensive description patterns in clinical observation, the finite element method has been reported as a useful tool to verify the fracture patterns and provide the spinal injury score [17]. A limitation of the Guo et al. study was the failure model of the PLC was simplified and not analyzed [17]. To eliminate the gray zone in the current TLICS, the PLC must be analyzed using the finite element method to evaluate the PLC status further when an injury is suspected or indeterminate (2 points, burst fracture-2 points, TLICS-4 points).

While both the Sterba et al. [18] and Wu et al. [19] studies analyze the PLC biomechanical properties, there is limited data on the finite element analysis evaluating the PLC status of indeterminate or injury suspected. A key component to eliminating the gray zone in the TLICS classification system is evaluating the PLC status to standardize and guide surgical treatment in patients with TL spinal injuries.

Based on the findings reported by Hsieh et al., finite element biomechanical analysis has demonstrated a useful technique for evaluating surgical treatment approaches of burst fractures [21]. The use of finite element modeling has proven to be an efficient tool to access postoperative outcomes by evaluating the biomechanics of hardware-related failures.

Biomechanics computer models of the spine have been developed using a wide range of approaches such as finite element models of various complexity. These models enhanced our understanding of the spine, especially with the increasing power of computers. The complexity of the human spine and variations in material properties and boundary conditions make it a suitable candidate for finite element modeling [21]. Moreover, the finite element method often provides significant advantages by providing a post-treatment assessment for spine injuries, such as TL burst fracture (TLBF), and where there are such individual variations, allowing cause-effect relationships to be isolated and thoroughly explored.

## Conclusions

This literature review suggested that the use of TLICS is safe, especially when treating single-column or three-column spinal injuries. Given that there is no universal acceptance of a classification system for TL injuries that helps to standardize approaches to treatment among interpreting physicians, the use of finite element analysis provides a useful tool to enhance the TLICS system. Special attention to TLICS application is necessary for the treatment of TLBF by eliminating the current gray zone. However, unstable TLBF inconsistencies in the total score among the TLICS and AO systems have led to a lack of standardization in surgical management. In patients with a TLICS score of 4, often from a burst fracture (2 points) and PLC injury suspected or indeterminate (2 points), their treatment plan is decided by the surgeon’s clinical judgment, leading to inconsistencies in the treatment approach among surgeons. Eliminating the gray zone will likely provide universal acceptance of a single classification system used by treating physicians, preventing discrepancies in scoring TLF. Finite element analysis offers a precise method to evaluate the PLC. However, limited studies have addressed uncertain PLC status. In such circumstances, an accurate assessment of neurological deficit plays an essential role.

Further studies using finite element analysis of TL spinal fractures would improve the TLICS classification system. Moreover, the analysis can provide a good understanding of post-treatment of several TL fracture patient types. For stable TL fractures, the biomechanical computational framework for accessing them quantified the effect of treatment aid in the evaluation of vertebral fractures and the understanding of factors contributing to fracture risk. Additionally, the biomechanical computational framework of unstable and stable TL fractures post-treatment using finite element analysis provided the most efficient tool to analyze the surgical hardware used and its long-term effect on the spinal system.
